# Transcutaneous auricular vagus nerve stimulation in the treatment of disorders of consciousness: mechanisms and applications

**DOI:** 10.3389/fnins.2023.1286267

**Published:** 2023-10-18

**Authors:** Likai Wang, Fei Gao, Zhan Wang, Feng Liang, Yongli Dai, Mengchun Wang, Jingyi Wu, Yaning Chen, Qinjie Yan, Litong Wang

**Affiliations:** ^1^Department of Rehabilitation Medicine, The Second Hospital of Dalian Medical University, Dalian, China; ^2^First Clinical Medical College, Shanxi Medical University, Taiyuan, China

**Keywords:** transcutaneous auricular vagus nerve stimulation, disorders of consciousness, neuroprotection, safety and tolerability, stimulation parameters, mechanisms of action

## Abstract

This review provides an in-depth exploration of the mechanisms and applications of transcutaneous auricular vagus nerve stimulation (taVNS) in treating disorders of consciousness (DOC). Beginning with an exploration of the vagus nerve’s role in modulating brain function and consciousness, we then delve into the neuroprotective potential of taVNS demonstrated in animal models. The subsequent sections assess the therapeutic impact of taVNS on human DOC, discussing the safety, tolerability, and various factors influencing the treatment response. Finally, the review identifies the current challenges in taVNS research and outlines future directions, emphasizing the need for large-scale trials, optimization of treatment parameters, and comprehensive investigation of taVNS’s long-term effects and underlying mechanisms. This comprehensive overview positions taVNS as a promising and safe modality for DOC treatment, with a focus on understanding its intricate neurophysiological influence and optimizing its application in clinical settings.

## 1. Introduction

Disorders of consciousness (DOC) are a group of conditions characterized by impaired awareness and arousal, characterized by impaired awareness and arousal, which typically result from severe brain injury, such as traumatic brain injury (TBI), stroke, and hypoxic–ischemic encephalopathy (Kang et al., 2022). Prolonged disorders of consciousness (pDOC) refer to pathological states of consciousness loss lasting more than 28 days, are classified by the patient’s altered state of consciousness as minimally conscious state (MCS) or unresponsive wakefulness syndrome (UWS), previously known as a vegetative state (*VS*) (Laureys et al., 2010; Scolding et al., 2021). UWS patients exhibit spontaneous eye opening but lack any signs of self or environmental awareness (Machado, 2002), while MCS patients display inconsistent but discernible signs of consciousness (Thibaut et al., 2021; Guo et al., 2022). Currently, the available opportunities for intervention for DOC include providing supportive care, managing complications, and optimizing the patient’s overall physical and neurological status (Edlow et al., 2021). Although pharmacological interventions (including dopaminergic and other neurotransmitter-modulating drugs), have been extensively used and achieved clinical success, these interventions tend to be inefficient and associated with side effects, when faced with coexistence of multiple medical conditions in DOC patients (Kondziella et al., 2020). Invasive brain stimulation techniques are implemented in deep brain to improve the accuracy, but also increase the risks of infection, as well as the risk of device malfunction (Raguž et al., 2021). Taking into account the limitations of interventions, non-invasive techniques [including transcranial magnetic stimulation (TMS) and transcranial direct current stimulation (tDCS)], as the safer ways and more focused approach to modulating key brain networks are applied (Formica et al., 2021; Guo et al., 2022; Han et al., 2022).

Invasive cervical vagus nerve stimulation (VNS) is a technique that involves implanting a device under the skin of the chest that delivers electrical impulses to the vagus nerve (VN) in the neck (Giordano et al., 2017). It has received approval by the US Food and Drug Administration (FDA) for the treatment of conditions such as epilepsy, depression, obesity, and as a modality for stroke rehabilitation (Milby et al., 2008; Giordano et al., 2017; Austelle et al., 2022; Hilz, 2022). However, the procedure necessitates surgery and can present a range of side effects, including voice changes, coughing, headaches, infections, and bleeding. Furthermore, it is costly and not universally accessible, making it an unsuitable option for some patients (Giordano et al., 2017). Recognizing these challenges, transcutaneous auricular vagus nerve stimulation (taVNS) was introduced as a non-invasive, more affordable, and easily implemented alternative. TaVNS is a non-invasive brain stimulation technique that involves the delivery of electrical stimulation to the auricular branch of the vagus nerve (ABVN) (Badran et al., 2018), accessible through the skin of the outer ear. Considerable evidence has suggested that taVNS is a therapeutic intervention for various neurological and psychiatric conditions, such as epilepsy (Thompson et al., 2021), depression (Liu et al., 2020), and migraine (Zhang et al., 2021). Regarding DOC, taVNS has the potential to modulate thalamo-cortical connectivity and neurotransmitter systems (Vitello et al., 2023), which are critical for promoting awareness and arousal. As a non-invasive method, taVNS avoids the risks associated with invasive procedures and offers a more focused approach to modulating key brain networks involved in consciousness, characterized by its bottom-up neural transmission pathway, when compared to other non-invasive stimulation techniques like TMS and tDCS (Wu et al., 2021).

As taVNS continues to gain rapid traction in both popularity and application for DOC, a consensus remains elusive in the field regarding the optimal timing for initial intervention, preferred stimulation sites, and the most effective stimulation parameters. The inquiry into whether taVNS can yield comparable restorative outcomes in DOC recovery, along with an exploration of the underlying mechanisms and prospects for future research directions, requires further exploration. Hence, the primary objective of this comprehensive review is to delve into the existing body of research on taVNS in the context of DOC, offering narrative insights into its potential therapeutic efficacy and the mechanisms through which it might engender its positive effects. The abbreviations in this review are listed in Table 1.

**Table 1:** List of abbreviations.

Abbreviation	Definition	Abbreviation	Definition
ABVN	The auricular branch of the vagus nerve	NE	Norepinephrine
ACh	Acetylcholine	Non-RtAS	Non-responded to auditory stimuli
BBB	Blood–brain barrier	NTS	Nucleus tractus solitarius
BDNF	Brain-derived neurotrophic factor	RtAS	Responded to auditory stimuli
CNS	Central nervous system	SN	Salience Network
CRS-R	Coma Recovery Scale-Revised	taVNS	Transcutaneous auricular vagus nerve stimulation
DMN	Default Mode Network	TBI	Traumatic brain injury
DOC	Disorders of consciousness	tVNS	Transcutaneous vagus nerve stimulation
EEG	Electroencephalography	UWS	Unresponsive wakefulness syndrome
EXN	External Network	VNS	Vagus nerve stimulation
HRV	Heart Rate Variability	VN	Vagus nerve
LC	Locus coeruleus	*VS*	Vegetative state
MCS	Minimally conscious state		

## 2. TaVNS principles and mechanisms

TaVNS apples application of electrical microcurrents to specific points on the auricle to stimulate the ABVN (Ben-Menachem et al., 2015). This approach offers a more accessible and minimally invasive alternative to traditional VNS methods (Marin et al., 2018). However, non-invasive VNS also has some limitations, such as lower stimulation intensity, lower specificity, lower reliability, and higher variability (Ahmed et al., 2022). And it’s worth noting that traditional VNS is still widely used because it has been shown to have greater efficacy for certain FDA-labeled conditions, including headache, epilepsy, and depression (Milby et al., 2008; Austelle et al., 2022; Hilz, 2022).

### 2.1. Auricular branch of the VN and stimulation techniques

The VN is a mixed nerve with approximately 80% afferent and 20% efferent axonal projections. The VN is mainly composed of sensory fibers, which can be classified by their conduction velocity and diameter of A-fibers (fastest), B-fibers (slower), and C-fibers (slowest) (Yuan and Silberstein, 2016). Aα fibers are notably the largest and have the fastest conduction, while unmyelinated C fibers are the smallest and demonstrate the slowest velocity. Aβ, Aγ, Aδ, and B fibers occupy an intermediate position in terms of both size and conduction speed. Crucially, during VNS in pigs—whose vagus nerve structure and size closely resemble that of humans—the recruitment of neuronal fibers is governed by their closeness to the stimulation electrode and the intensity of the local electric field. There is an inverse relation between fiber size and its recruitment sequence, leading to A-type fibers being recruited before C-type fibers (Settell et al., 2020).

The cervical branch vagus nerve is made up of about 20% myelinated A and B fibers and 80% unmyelinated C fibers (Vonck et al., 2009). However, research to elucidate the mechanism of action of VNS has shown that effective stimulation in humans is primarily mediated by afferent vagal A- and B-fibers (Evans et al., 2004; Howland, 2014). According to several studies, the destruction of peripheral C-fibers does not alter subsequent VNS-induced seizure suppression in rats. Based on a particular study (Mourdoukoutas et al., 2018), with stimulation intensities aligned to clinical nVNS protocols and a current of 10 mA, there was a preferential activation of A-fibers and large B-fibers, while C-fibers remained unaffected. These results indicate that activation of vagal C fibers is not necessary for VNS-induced seizure suppression. However, effective therapeutic stimulation parameters appear to be subthreshold for these fibers in humans, and there are no clinical reports of the autonomic side effects that would be expected if these fibers were maximally activated (Krahl, 2012). The anatomic basis for non-invasive VNS relies on the fact that some branches of the VN have cutaneous distribution, meaning that they can be accessed through the skin. The external ear receives its complex sensory innervation from multiple nerves, including the auricular branch of the vagus, auriculotemporal nerve, greater auricular nerve, and lesser occipital nerve. Research on ear anatomy reveals that the tragus, concha, and cymba concha are sites on the human physique where there’s a presence of cutaneous afferent vagal nerve pathways (Peuker and Filler, 2002). The cymba conchae region of the external ear known as the auricle is the unique area for stimulating vagus nerve (VN). The distribution of the auricular branch throughout the auricle is nearly 100%. It is the only surface area of the body where the ABVN is distributed, making it an ideal target for taVNS (Yakunina et al., 2017).

The ABVN primarily consists of myelinated Aβ fibers, myelinated Aδ fibers, and unmyelinated C fibers (Bermejo et al., 2017). The number of Aβ fibers is comparable between the left and right ABVN (Safi et al., 2016). Notably, the ABVN has approximately five to six times fewer Aβ class nerve fibers compared to the cervical branch of the VN (Safi et al., 2016). There’s considerable individual variation in fiber count, complicating the determination of optimal stimulation sites and parameters for effective tVNS using the ABVN. Detailed examination of nerve fiber types within the ABVN, suitable for computational modeling, is lacking. As such, the understanding of the unique nerve fiber types remains somewhat speculative. Evaluations of therapeutic effectiveness largely rely on observed therapeutic benefits, further correlated with primary and secondary outcomes. In general, the VN is pivotal in brain–body interactions, sparking interest in its artificial stimulation for therapeutic applications.

### 2.2. Modulation of vagal activity and its therapeutic implications

The VN’s afferent fibers terminate in multiple regions of the brainstem, including the nucleus ambiguus, nucleus dorsalis of the vagus, nucleus tractus solitarius (NTS), and spinal trigeminal nucleus (Wang et al., 2014). The NTS mainly receives and integrates these fiber signals and projects to other areas, which leads to changes in the release of significant neurotransmitters such as norepinephrine (NE), acetylcholine (ACh), serotonin, and dopamine (Hulsey et al., 2017; Zhang et al., 2019). The VN serves as a pivotal conduit between the brain and numerous bodily organs, transmitting both sensory and motor signals. It plays a fundamental role in the autonomic regulation of vital functions, including heart rate, blood pressure, respiration, and digestion (Mazzone and Undem, 2016; Waise et al., 2018). Furthermore, the VN also forms a link between the gut and the brain, known as which may influence conditions like obesity, epilepsy, and depression (Breit et al., 2018; Zhang et al., 2020). These demonstrate that taVNS impacts vagal activity through complex mechanisms and could be a potential treatment strategy for a variety of medical conditions involving the modulation of neuroplasticity, inflammatory responses, and the balance of sympathetic and parasympathetic activity.

NTS is the primary relay between the VN and the central nervous system (CNS), with projections extending to the cortex. Through direct or indirect anatomic connections via the NTS, VNS alters the activity of many cortical and subcortical regions (Breit et al., 2018). The consciousness and awareness networks such as the Default Mode Network (DMN) and the Salience Network (SN) are closely related. Recent studies have suggested that taVNS modulates or activates the cerebral cortices and subcortical areas that are related to the control of consciousness. The central projection of the ABVN is consistent with the classic central vagal projection, which includes the NTS, the locus coeruleus (LC), and the nucleus ambiguous (Knowles and Aziz, 2009; Butt et al., 2020). Then stimulation of the ABVN can activate these nuclei and influence their downstream targets, such as the amygdala, hippocampus, thalamus, and prefrontal cortex.

The DMN plays a foundational role in introspective and self-referential activities, with key areas including the medial prefrontal cortex (mPFC) and the posterior cingulate cortex/precuneus. And the SN is crucial for detecting and directing attention to salient stimuli, primarily involves the anterior cingulate cortex (ACC) and the insula. A study illustrated that the connectivity between the DMN and Central Executive Network (CEN) oscillates in response to taVNS frequencies (Rong et al., 2016): 1 Hz taVNS led to diminished DMN-CEN connectivity, while 25 Hz enhanced it. Moreover, both frequencies conspicuously bolstered the DMN-SN connectivity, elucidating the intricate interplay between introspection and attention processing (Li et al., 2016; Yakunina et al., 2017). NE exerts a range of functional effects in the CNS, especially in regulating alertness, attention, stress responses, and the sleep–wake cycle. Stimulation of the VN has been shown in some studies to activate the LC, leading to an increase in NE release (Hulsey et al., 2017; Jacobs et al., 2020; Farrand et al., 2023). Besides, ACh is fundamental to the brain’s arousal systems, with its release being intrinsically linked to heightened arousal and alertness. VNS has been shown to induce the release of ACh in the brain (Meyers et al., 2019). The release of neurotransmitters ACh and NE widely activate the brain’s neuromodulatory network, enhancing neural plasticit (Hays et al., 2013). Those are the neurological basis for exploring the therapeutic potential of taVNS to modulate levels of consciousness. However, more research is needed to fully understand the complex pathways involved in the projections of the VN to the cortex.

## 3. Mechanisms of VNS in animal experiments for consciousness disorders

VNS in animal experiments has shown potential in consciousness disorders through its effects on reducing cell apoptosis, modulating neurotransmitters, decreasing inflammatory responses, and preserving the integrity of the blood–brain barrier (BBB).

### 3.1. Reducing cell apoptosis

Research has shown that VNS can have positive effects on the brain’s health to reduce cell apoptosis in animal models through various mechanisms, which include the regulation of miR-210, appetite-regulating hormones, the PI3K/AKT signaling pathway, and brain-derived neurotrophic factor (BDNF) (Follesa et al., 2007; Dong and Feng, 2018; Lai et al., 2019).

Jiang et al. identified miR-210, caused by VNS, as a regulator of neuronal apoptosis in a mouse model of hypoxic–ischemic brain injury (Jiang et al., 2015). Zhao et al. also found that neuronal apoptosis significant decrease after VNS in a rat model of cerebral ischemia–reperfusion injury (Zhao et al., 2019). Considerable evidence has suggested that VNS affect cell apoptosis through the pathways mentioned above, which are associated with reducing the size of an infarct and promoting the survival of neurons.

### 3.2. Modulating neurotransmitters

VNS can regulate the expression of both excitatory (upregulate) and inhibitory (downregulate). This dual modulation may be the underlying mechanism for its arousal-promoting effects.

Several studies have indicated that the upregulate of excitatory neurotransmitters are particularly noticeable in brain regions associated with wakefulness (including the prefrontal cortex and hypothalamus which involved in sleep regulation) (Dong and Feng, 2018; Li et al., 2018) by boosting neuronal activity and promoting wakefulness. The reduction of inhibitory neurotransmitters resulting from VNS, such as gamma-aminobutyric acid (GABA) and serotonin, which are involved in sleep regulation, could promote arousal and wakefulness (Manta et al., 2013; Schwartz and Kilduff, 2015; Sanders et al., 2019).

### 3.3. Decreasing inflammatory response

VNS has a significant impact on regulating immune response and controlling inflammation through the cholinergic anti-inflammatory pathway.

This process consists of the CNS receiving inflammatory signals (Zachs et al., 2019), transmitted through efferent fibers from the dorsal motor nucleus of the VN sending anti-inflammatory signals to the spleen (Huston and Tracey, 2011; Stavrakis et al., 2015). At this point, peripheral nerve terminals in the spleen release NE (Hilderman et al., 2015), which activates adrenergic receptors on specific T lymphocytes. This activation triggers the expression of acetyltransferase and leads to the synthesis of ACh. After the synthesis of ACh, it acts on macrophages that express the nicotinic ACh receptor (α7nAChR) (Johnston and Webster, 2009; Calvillo et al., 2011; Wang et al., 2012; Wang J. Y. et al., 2021; Caravaca et al., 2022), suppressing the production and release of pro-inflammatory cytokines and promoting the release of anti-inflammatory cytokines (Lei and Duan, 2021; Chen et al., 2022). VNS is a highly effective way to regulate the activity of immune cells, leading to a significant reduction in inflammation. This makes it an invaluable tool in improving outcomes for individuals with consciousness disorders.

### 3.4. Lowering blood–brain barrier permeability

The integrity of the BBB plays crucial role in maintaining brain homeostasis and shielding the brain from harmful substances (Hugon et al., 2021). Disruption of the BBB following brain injury can lead to increased permeability, allowing the infiltration of inflammatory cells and molecules into the brain parenchyma, exacerbating neuroinflammation and neuronal damage (Åslund et al., 2017). VNS holds promise for reducing the permeability of the BBB following brain injuries, potentially mitigating neuroinflammation, preserving neuronal function, and thereby improving outcomes in consciousness disorders.

VNS’s impact on BBB permeability is believed to stem from several mechanisms (Lopez et al., 2012; Chen et al., 2018). One of the mechanism involves the regulation of tight junction proteins, such as occludin and claudin (Chen et al., 2020). Research indicates that VNS can increase the expression of these tight junction proteins, thereby enhancing the barrier function of the BBB and reducing its permeability to inflammatory factors (Lopez et al., 2012; Chen et al., 2018). Furthermore, studies have demonstrated that VNS can inhibit the activity and expression of MMPs, thereby attenuating BBB disruption and reducing the entry of inflammatory cells and molecules into the brain (Yang et al., 2018; Li et al., 2020).

## 4. Application and evaluation of taVNS in disorders of consciousness

Through the analysis of behavioral and neurophysiological outcomes, a deeper understanding of the effects of taVNS on brain activity and behavioral response in DOC patients is achieved. By contrasting the efficacy of taVNS across various patient categories, including *VS*/UWS and MCS, differences in outcomes are revealed for diverse types and etiologies of consciousness disorders. This analysis assists in identifying the most effective application schemes for taVNS therapy.

### 4.1. Assessment methods and neurophysiological outcomes of taVNS treatment

In order to investigate the impact of behind taVNS on consciousness recovery, a combination of behavioral and neurophysiological evaluations are employed, including the Coma Recovery Scale-Revised (CRS-R), electroencephalography (EEG), and functional magnetic resonance imaging (fMRI). These methodologies shed light on the influence of taVNS on brain activity, connectivity, and behavioral responsiveness in patients with DOC.

#### 4.1.1. Behavioral assessment: CRS-R

The CRS-R, a behavioral scale that assesses responsiveness across auditory, visual, motor, communication, and arousal domains, is frequently used to investigate taVNS’s efficacy in promoting consciousness recovery (Di et al., 2017). Despite the observed improvements in CRS-R scores for both UWS and MCS patients, additional research is necessary to pinpoint the specific factors driving these enhancements and understand the relationship between CRS-R scores and neurophysiological changes.

#### 4.1.2. Electroencephalography

Electroencephalography provides insights into changes in brain activity following taVNS treatment. However, its usage in DOC patients is sometimes limited by artifacts and the necessity for a more standardized approach to data interpretation. Future enhancements might include high-density EEG for higher spatial resolution and improved signal quality, as well as improved algorithms for artifact reduction and data processing (Kondziella et al., 2020).

EEG-derived biomarkers, including EEG background activity, reactivity, event-related potentials (ERPs), and spectral power, are essential for diagnosing and predicting recovery in patients with DOC (Edlow et al., 2021). Among these biomarkers, reactive alpha rhythms in DOC patients indicate intact neural processing and the operational efficacy of crucial brain networks, especially the thalamocortical circuits (Porcaro et al., 2021). Reactive alpha rhythms can also help distinguish between levels of consciousness in DOC, with MCS patients typically showing more preserved alpha activity than those in *VS* or UWS (Bai et al., 2021). ERPs from EEG offer insights into the brain’s particular responses to external stimuli, with components like the P300 serving as windows into the cognitive functioning of DOC patients (Rollnik, 2019). Studies suggest that the combination of EEG-based connectivity analysis and ERP analysis can significantly improve the detection of consciousness recovery after taVNS treatment.

#### 4.1.3. Functional magnetic resonance imaging

In patients with DOC, fMRI can investigate altered brain network connectivity, highlighting changes in the DMN and other primary networks both before and after taVNS treatment, signifying the potential restoration of functional networks. Although studies have demonstrated the fMRI tend to expensive and inaccessibility, more efforts can be taken to explore different types of fMRI (resting-state and task-based) and offer more comprehensive understanding of the neural mechanisms involved in taVNS treatment (Kondziella et al., 2020; Snider and Edlow, 2020).

#### 4.1.4. Integration of imaging techniques

Combining fMRI with other neuroimaging techniques like diffusion tensor imaging (DTI) or magnetoencephalography (MEG) could provide a broader perspective on the structural and functional changes following taVNS treatment (Pourmotabbed et al., 2022).

#### 4.1.5. Emerging neuroimaging techniques: near-infrared spectroscopy

Near-Infrared Spectroscopy (NIRS), an upcoming neuroimaging technique, has potential to offer insights into the neural mechanisms underlying taVNS treatment and its effects on consciousness recovery. Although NIRS has advantages, further research is required to validate its reliability in evaluating the effect of taVNS on brain function in DOC patient (Si et al., 2018; Zhang et al., 2018).

### 4.2. Comparative analysis of taVNS effects in DOC patients

#### 4.2.1. Effects of taVNS in *VS*/UWS and MCS patients

Several studies have investigated the effectiveness of taVNS in promoting consciousness recovery in patients with *VS*/UWS. Generally, these studies have utilized the CRS-R as the primary outcome measure to assess changes in patients’ consciousness levels. For example, Yu ‘s case report demonstrated significant improvement in CRS-R scores for a 73-year-old female patient in a *VS* for 50 days following cardiopulmonary resuscitation after receiving taVNS treatment (Yu et al., 2017). In the case study by Osińska et al., (2022), a 28-year-old female with persistent UWS due to TBI for 6 years was examined. Behavioral indices of consciousness improved during taVNS but ceased post-stimulation. Additionally, EEG analysis demonstrated increasing alpha power over months of taVNS, suggesting possible neural reintegration.

Studies suggested that taVNS is a promising therapy for MCS patient. For instance, Noé et al. study observed significant improvements in CRS-R scores, particularly among MCS patients (Noé et al., 2020). Zhou conducted a randomized controlled trial and discovered that there were noticeable variations in CRS-R scores between the active taVNS and sham groups for patients in a state of minimal consciousness, but there were no significant differences in scores for patients in a *VS* (Zhou et al., 2023). Wang’s RCT in China enrolled 12 participants (6 with MCS and 6 with *VS*). The study unveiled distinct modulatory effects of taVNS on consciousness states. Particularly, taVNS exhibited selective influence on delta wave energy and brain connectivity. Importantly, MCS patients displayed a notable taVNS response, showcasing heightened brain connection activity that could potentially facilitate awakening.

#### 4.2.2. Effects of taVNS in TBI-related and non-TBI-related DOC

It is necessary to explore the therapeutic effect of taVNS on disturbance of consciousness caused by TBI and not-TBI, respectively. There may be different responses to taVNS depending on the etiology of the brain injury, such as TBI and not-TBI. For example, tbi may cause more diffuse axonal injury and damage to the white matter tracts, while non-TBI may cause more focal lesions and ischemia (Briand et al., 2020). These differences may affect the neural pathways and networks that are modulated by taVNS.

Studies on TBI-related DOC patients have shown promising results regarding the effectiveness of taVNS in promoting consciousness recovery. Hakon’s feasibility study on patients with severe TBI reported progress in the consciousness state of three patients (Hakon et al., 2020). Wang’s RCT found that taVNS might improve brain connectivity, particularly in MCS patients with TBI (Yifei et al., 2022).

For non-TBI-related DOC patients, taVNS were found as potential strategy for promoting consciousness recovery. It has been discovered that patients who still have normal hearing abilities, particularly those who are not suffering from traumatic brain injuries, tend to have a more positive response to taVNS therapy. Additionally, taVNS has been observed to increase blood flow in various regions of the brain for these patients (Yu et al., 2021).

#### 4.2.3. Effects of taVNS in RtAS and non-RtAS DOC

In the exploration of taVNS effects within the realm of DOC, Yu’s study from China focused on patients categorized under responded to auditory stimuli (RtAS) and non-responded to auditory stimuli (non-RtAS) subtypes. The study encompassed 10 participants, comprising 7 in *VS* and 3 in MCS. Notably, the study revealed favorable outcomes following 4 weeks of taVNS treatment in RtAS DOC patients, while non-RtAS cases exhibited dissimilar responses. The influence of taVNS was further elucidated by elevated cerebral blood flow in diverse brain regions among RtAS DOC patients, with a notably restricted impact in non-RtAS individuals. Remarkably, the presence of preserved auditory function emerged as a pivotal determinant of taVNS responsiveness in DOC patients. These findings suggest the potential of taVNS to ameliorate RtAS DOC conditions through engagement with pivotal neural networks, including the salience network, limbic system, and interoceptive system. The study’s insights illuminate the contrasting taVNS effects observed within disorders of consciousness, effectively distinguishing between RtAS and non-RtAS instances.

## 5. The impact of taVNS on consciousness in DOC

### 5.1. Brain networks involved in consciousness control

Understanding the neural mechanisms controlling consciousness is crucial for developing therapeutic strategies for DOC. While the complete understanding of the mechanisms involved is still unclear, several key brain networks and their interactions are involved in the regulation of consciousness.

These networks include: Ascending Reticular Activating System (ARAS): The ARAS originates in the upper brainstem and plays a key role in arousal, wakefulness, and alertness (Duclos et al., 2020). It connects with the central thalamus and cerebral cortex, providing neuronal input to other networks involved in consciousness; Forebrain Circuit: This cortico-striato-thalamo-cortical loop interacts with the ARAS, frontal lobe, and central thalamus, contributing to the regulation of forebrain arousal (Li et al., 2012); Frontoparietal Network: Comprising two subnetworks, DMN and the External Network (EXN), the frontoparietal network is responsible for internal consciousness, attention, and action selection (Amgalan et al., 2022); SN: The SN connects various regions of the brain and plays a switching role between the DMN and EXN, facilitating the regulation of consciousness (Li et al., 2017).

Understanding the roles of these networks in consciousness control is crucial for developing effective treatment methods for DOC, including taVNS. In the context of taVNS therapy, these networks serve as potential targets for inducing recovery of impaired consciousness in DOC patients.

### 5.2. Pathophysiology of UWS and MCS

The pathophysiological mechanisms of different consciousness disorders may vary, and understanding these differences may aid in developing more targeted and effective treatment methods. Research indicates that patients with UWS and MCS exhibit significant changes in the frontoparietal and DMNs, leading to differences in their levels of consciousness (Escrichs et al., 2022).

UWS patients display severe disruption of connectivity within the frontoparietal network and between the frontoparietal network and the DMN (Zhou et al., 2019). This disruption is associated with a lack of conscious awareness and response to external stimuli. Moreover, UWS patients exhibit reduced activity and connectivity in the DMN, which is related to a lack of self-referential processing and internal consciousness (Dong et al., 2018).

Compared to UWS patients, MCS patients showed partially preserved connections in the frontoparietal network and DMN (Farahani et al., 2019). Although these connections may be weaker than in healthy individuals, they are sufficient to support minimal levels of consciousness and responses to external stimuli (Fernández-Espejo et al., 2012; Crone et al., 2014). MCS patients exhibit higher activity in the DMN compared to UWS patients, indicating a degree of preserved self-referential processing and internal consciousness.

Understanding these differences in neural network connectivity and activity in UWS and MCS patients may inform the development of tailored treatment methods, including taVNS. By targeting specific disruptions in connectivity and activity within these networks, it may be possible to enhance the effectiveness of taVNS in promoting consciousness recovery in patients with different consciousness disorders.

### 5.3. Mechanisms of taVNS on consciousness recovery in DOC

The mechanisms of action of taVNS involve complex interactions between neural networks and functions. It has a comprehensive impact on brain network connectivity, neurotransmitters, neuronal excitability, neural plasticity, and inflammation, which may collectively contribute to the recovery of impaired consciousness in patients with DOC.

#### 5.3.1. Brain network connectivity and neurotransmitters

Activation of the spinal trigeminal nucleus and NTS (Zhao et al., 2017): taVNS stimulates the ABVN, leading to the activation of the spinal trigeminal nucleus and NTS in the lower brainstem. This activation plays a crucial role in the arousal process and regulates various functional aspects of consciousness networks.

Activation of the reticular activating system (RAS) in the LC and raphe nuclei (Sumi-Akamaru et al., 2016): The activated NTS influences the LC and raphe nuclei in the upper brainstem, part of the RAS. This activation contributes to arousal and alertness and is involved in neurotransmitter pathways, including the noradrenaline and serotonin pathways.

Regulation of the thalamus, striatum, and central loop model: The LC (noradrenaline pathway) and raphe nuclei (serotonin pathway) directly activate the thalamus, which in turn activates the striatum (Osińska et al., 2022). This activation helps reconstruct the central loop model, promoting consciousness recovery through the support of forebrain arousal regulation.

Activation of the SN, EXN, and DMN (Feng et al., 2022): Activation of the LC (noradrenaline pathway) influences consciousness by promoting SN and EXN activation, involved in attention and action selection. SN activation helps switch from DMN to EXN, improving negative connectivity between DMN and EXN, which is essential for internal awareness and self-referential processes.

Activation of the raphe nuclei and DMN connectivity (Rong et al., 2016): Activation of the raphe nuclei (serotonin pathway) can increase DMN activity and connectivity, which may help improve consciousness in DOC patients.

#### 5.3.2. Effects on neural plasticity

TaVNS has been found to promote neural plasticity, the brain’s ability to reorganize and form new neural connections. Studies have shown that taVNS can enhance long-term potentiation and increase the expression of neurotrophic factors, such as BDNF (Wang Y. M. et al., 2021). This is particularly important for DOC patients, as it can promote the recovery of damaged neural pathways and the reconstruction of more functional neural circuits. Significantly, taVNS is believed to influence neural activity through the global release of neuromodulators in the brain.

The cholinergic system is primarily characterized by the neurotransmitter ACh and has diverse effects throughout the body, especially in the CNS. The fundamental principle behind VNS-evoked cortical neuromodulation is to amplify the positive effects of VNS by coupling its activation with particular sensory or motor events. By temporally associating this neuromodulatory effect with distinct sensory or motor events, synaptic connections linked to those events are fortified, thus encouraging long-term potentiation and increased neural plasticity (Hays et al., 2013). Research indicates a significant contribution of VNS-evoked basal forebrain ACh release in the neocortex (Mridha et al., 2021). From a motor perspective, VNS paired with forelimb movements instigates reorganization within the motor cortex, but notably, this plasticity does not arise if lesions develop in the cortical cholinergic projections originating from the basal forebrain (Hulsey et al., 2016). On the sensory front, coordinating sound exposure with VNS induces a reshaping of the auditory cortical map, mirroring the alterations observed when sounds are synchronized with basal ganglia stimuli (Borland et al., 2019).

The maintenance of auditory function might play a crucial role in the responsiveness of patients with DOC to taVNS. This is supported by findings indicating that in patients who did not exhibit a response to auditory stimulation, the surge in cerebral blood flow during taVNS treatment was notably subdued (Yu et al., 2021). On another front, the effect of VNS on cortical excitatory synchrony diminishes when muscarinic receptors in the auditory cortex are inhibited, suggesting a significant role for ACh released from the basal forebrain (Engineer et al., 2011; Nichols et al., 2011). Further research (Mridha et al., 2021) has determined that the response of auditory cortical neurons to VNS is dependent on stimulation parameters, with varying degrees of activation in cholinergic neurons based on different stimulation settings. Moreover, this research also discovered that the pupil dilation is induced by cholinergic fibers triggered by VNS, and its behavior varies based on the immediate state of the brain. This suggests pupil dilation as a potential non-invasive biosensor for VNS-induced alterations in neuromodulatory brain states.

#### 5.3.3. Neuronal excitability regulation

TaVNS can directly or indirectly influence neuronal excitability by stimulating the VN (Zheng et al., 2021). It may modulate the activity of ion channels and neurotransmitter systems, leading to changes in the excitability of neural circuits. By modulating neuronal excitability, taVNS may help restore the normal function of affected neural circuits, thereby improving cognitive and behavioral outcomes for DOC patients.

#### 5.3.4. Anti-inflammatory effects

Inflammation is often associated with various neurological disorders, including brain injury and neurodegenerative diseases. TaVNS has been shown to have anti-inflammatory effects by modulating the release of cytokines and other inflammatory mediators (Rawat et al., 2019). By reducing inflammation, taVNS can promote a more favorable environment for neural recovery in DOC patients, which may ultimately contribute to improvements in consciousness and cognitive function.

#### 5.3.5. Autonomic nervous system regulation

TaVNS has been shown to regulate the activity of the autonomic nervous system, particularly the balance between the sympathetic and parasympathetic branches (Clancy et al., 2014; Colzato et al., 2018). Stimulating the VN can increase parasympathetic activity, which can indirectly impact consciousness recovery by promoting overall brain health and function. Additionally, activation of the VN can regulate the release of neurotrophic factors, such as BDNF, which can promote neural plasticity and aid in the recovery of consciousness.

In conclusion, the mechanisms of action of taVNS on consciousness recovery involve the activation of vagal nerve cortical pathways, neurotransmitter regulation, and autonomic nervous system regulation. These mechanisms can collectively promote the restoration of functional networks that support consciousness, ultimately leading to improvements in behavioral and neurophysiological outcomes. Further research is needed to better understand the specific interactions and contributions of these mechanisms in the context of taVNS treatment for DOC.

## 6. Factors influencing treatment response

In administering taVNS treatment, it becomes clear that a one-size-fits-all approach is insufficient. The unique conditions and recovery goals of each patient demand that we adjust the stimulation parameters accordingly. Especially in the case of consciousness disorders, precision therapy is the crux of effective treatment. The delicate balance among stimulation parameters hinges on the complex interplay of the patient’s condition, recovery goals, and the biophysical characteristics of the nerve fibers. This intricate dance holds immense potential for unlocking new pathways to restore consciousness in patients with consciousness disorders. In Table 2, a summarized overview is provided, detailing the stimulation sites, device characteristics, parameter settings, stimulation duration, main findings, and side effects from relevant studies.

**Table 2:** Stimulation location, parameters, therapeutic effects, and side effects for all studies assessing the efficacy of taVNS in patient with DOC.

First author	Study groups	No. of disorders	Stimulation sites and device	Parameter settings	Stimulation Period	Assessment	Clinical outcomes	Side effects
Zhou, 2023	taVNS/sham group; RCT	*VS*:29; MCS:28	Left outer ear; tVNS 501 Changzhou Rishena Medical Device Co., Ltd., Jiangsu, China	20 Hz, 200us	4 weeks, 30 min/session	CRSR	Effective for MCS, not *VS*/UWS	Common side effects, unrelated to taVNS.
Osińska, 2022	Case Report	UWS for 6 years, TBI, 28-year-old female	tVNS Technologies, Erlangen, Germany	25 Hz, 250us, single-phase square wave, 30s-30s, 0.2 mA-1.5 mA	6 months; every day; 4 h/day	CRSR; EEG	Behavioral consciousness indices rose with tVNS, halted post-stimulation. EEG alpha power increased over months of tVNS, suggesting neural reintegration.	None reported
Wang, 2022	taVNS/sham group; RCT	*VS*/UWS:7; MCS:5	Bilateral auricular concha; Huatuo brand electronic acupuncture instrument (SDZ- II B type, Suzhou Medical Products Factory Co.)	20 Hz, <1000us, 4-6 mA	14 days, twice per day for four consecutive weeks, 30 min in each ear	CRSR; EEG	Differentially modulates consciousness states; Effective for MCS; Alters delta wave energy and brain connectivity selectively.	None reported
Vitello, 2022	taVNS/sham group; RCT	TBI 7–90 days	Bilateral over the cymba conchae; Cerbomed GmbH, Germany, NEMOS/tVNS®	25 Hz, 200-300us, 30s-30s, 3 mA	90 days, 45 min/day	CRSR, hd-EEG	None reported	None reported
Yu, 2021	RtAS/nRtAS; RCT	MCS:3; *VS*: 7	Over the concha	20 Hz, 500 ms, 4-6 mA	30 min; twice a day (8:00 and 16:00) for 4 weeks	CRSR; 3.0 T ASL-FMRI	Boosted CBF in RtAS, not in nRtAS.	None reported
Noé, 2020	RCT	*VS*/UW: 6; MCS: 8	Left tragus; Parasym® CE	20 Hz, 250us, 1.5 mA, Sinusoidal waveform	30-min sessions, twice a day (5 days per week), 40 sessions	CRSR	MCS patients respond better than *VS*/UWS patients; VNS benefits emerge with time and repeated treatment.	None reported
Hakon, 2020	Single-Armed Open-Label Feasibility Study	TBI: *VS*/UWS:3; MCS:2	Left cymba conchae; tVNS Nemos® (Cerbomed, Germany; CE-marked 2011)	25 Hz, 250us, 30 s on/ 30 s off, with up to 0.5 mA for the first 3 days, and subsequently 1 mA for the remaining eight-week period	8 weeks	CRSR	tVNS for 8 weeks, 4 h daily, is feasible and safe in *VS*/UWS and MCS patients	None reported
Yu, 2017	Case Report	*VS* for 50 days after cardiopulmonary resuscitation, 73-year-old female	Bilateral ear concha	20 Hz, <1000us, 4-6 mA	Twice daily for 30 min each, for four consecutive weeks	CRSR; 3.0 T fMRI	Shift from *VS*-MCS associated with altered brain FC patterns taVNS promotes enhanced functional connectivity of DMN.	None reported

### 6.1. Stimulation parameters

In taVNS therapy, the choice of stimulation parameters is critical as it directly impacts the treatment outcomes. The primary stimulation parameters include the waveform, pulse width, intensity, frequency and duration of the stimulus.

#### 6.1.1. Frequency

Four studies adopted a frequency of 20 Hz (Yu et al., 2017, 2021; Yifei et al., 2022; Zhou et al., 2023). On the other hand, four other studies each utilized a slightly higher frequency of 25 Hz (Hakon et al., 2020; Noé et al., 2020; Osińska et al., 2022; Vitello et al., 2023). The convergence around the 20 Hz to 25 Hz range indicates a potential sweet spot in frequency parameters for taVNS, yet the nuances of these choices underscore the continued efforts to fine-tune the therapy for optimal results.

While the FDA has approved 20–30 Hz frequencies for VNS (Groves and Brown, 2005), frequencies of 50 Hz and above may cause considerable and irreversible harm to the VN (Agnew and McCreery, 1990). Given the anatomical distinctions between the ABVN and neck VN, the safe frequency range for taVNS remains undefined. Thus, it’s incorrect to assume that the 20–30 Hz range is optimal for taVNS. Comprehensive controlled studies are essential to determine the impacts of different stimulation frequencies. Some researchers have already unveiled their study protocols in which patients undergo taVNS at five distinct frequencies (1, 10, 25, 50, and 100 Hz) to pinpoint the most effective stimulation frequency for treating DOC patients (Zhai et al., 2023).

#### 6.1.2. Pulse width

When considering taVNS parameter settings for the treatment of DOC, different studies have chosen varied pulse widths. One study used a pulse width of 200us (Zhou et al., 2023), aligning with the lower range of another that ranged from 200 to 300us (Vitello et al., 2023). Three other studies standardized their pulse width at 250us (Hakon et al., 2020; Noé et al., 2020; Osińska et al., 2022). Another RCT reported a pulse width of 500us (Yu et al., 2021), which is consistent with a broader parameter of less than 1000us observed in two other studies (Yu et al., 2017; Yifei et al., 2022). These varied choices reflect the diverse approaches researchers are taking in their quest to optimize therapeutic efficacy in DOC patients.

The choice of pulse width plays a pivotal role in determining the types of nerve fibers being activated (Rawat et al., 2019; Sclocco et al., 2020). For instance, short pulse width stimulations (such as 10 microseconds) are more likely to activate Aβ fibers, primarily associated with tactile transmission. On the other hand, longer pulse widths (e.g., 1,000 microseconds) are more inclined to stimulate various types of nerve fibers, including Aδ and C fibers related to pain perception (Salchow-Hömmen et al., 2018). Therefore, when selecting the pulse width, one must consider the target of stimulation and the anticipated sensory effects.

#### 6.1.3. Waveform

In taVNS studies for DOC treatment, one used a single-phase square wave (Osińska et al., 2022), while another adopted a sinusoidal waveform (Noé et al., 2020). Notably, several other studies did not specify their choice of waveform, suggesting that device-specific waveforms may have been used in these instances (Yu et al., 2017, 2021; Hakon et al., 2020; Yifei et al., 2022; Vitello et al., 2023; Zhou et al., 2023). The variations and omissions in waveform descriptions underscore the need for more standardized reporting in future research to fully understand the potential nuances and effects of different waveforms in taVNS treatment.

The choice of waveform can also influence the stimulation effects. For example, peak waveform stimulations could be more precise as some types of nerve fibers (like Aβ fibers) show heightened sensitivity to such waveforms (Song et al., 2020). In this scenario, a shorter pulse width can activate Aβ fibers more effectively, leading to a more accurate stimulation effect.

#### 6.1.4. Intensity

In the realm of taVNS treatments for DOC, various stimulation intensities are observed across studies. One study employed an intensity range from 0.2 mA to 1.5 mA (Osińska et al., 2022), another maintained a steady 1.5 mA (Noé et al., 2020), while a different research set the intensity at 3 mA (Vitello et al., 2023). Both an RCT and another study settled on an intensity span of 4–6 mA (Yu et al., 2021; Yifei et al., 2022), with a similar range seen in a separate case report (Yu et al., 2017). A distinct study adopted a more incremental strategy (Hakon et al., 2020), starting with an intensity of up to 0.5 mA for the initial 3 days and then elevating it to 1 mA for the subsequent eight-week duration. This variation in intensities across studies underscores the ongoing exploration to determine the optimal parameters for therapeutic efficacy in DOC patients.

The selection of stimulation intensity and frequency should be adjusted according to the patient’s pain threshold, aiming to achieve effective nerve stimulation without causing excessive pain. For patients with consciousness disorders, their perception of pain induced by electrical stimulation may be reduced due to sensory and cognitive limitations (Wang J. Y. et al., 2021). Therefore, in certain cases, it may be necessary to employ longer stimulation pulse widths and greater stimulation intensity to activate a variety of nerve fiber types. In the practice of neuroelectric stimulation, the choice of stimulated nerve fiber type (such as C fibers or Aβ fibers) is indeed crucial to the stimulation effect and therapeutic goals (Huang et al., 2018). This decision depends on the specific therapeutic objectives and the patient’s condition. Utilizing a larger pulse width may stimulate a variety of nerve fiber types concurrently, including motor and sensory nerves (Li et al., 2014).

#### 6.1.5. Duration of treatment

In taVNS treatments for DOC, the duration and regularity of sessions might influence therapeutic outcomes. One study opted for a 4-week protocol with 30-min sessions (Zhou et al., 2023), while another pursued a rigorous 6-month regimen with daily 4-h sessions (Osińska et al., 2022). An approach spanning 14 days involved two daily treatments for four consecutive weeks, dedicating 30 min to each ear (Yifei et al., 2022). Another protocol was structured over 90 days with sessions lasting 45 min each (Vitello et al., 2023). Several studies adopted a regimen of twice-daily sessions, each lasting 30 min (Yu et al., 2017; Noé et al., 2020; Yu et al., 2021). Of these, one lasted 4 weeks (Yu et al., 2021), while another covered 40 sessions over 5 days a week (Noé et al., 2020). A different study had an 8-week duration (Hakon et al., 2020). These variations underscore the diverse strategies in play, as researchers navigate the most effective treatment duration and frequency for taVNS in DOC patients.

### 6.2. Stimulation site

In taVNS treatments for DOC, studies have explored various ear locations for stimulation. One study targeted the left outer ear (Zhou et al., 2023), while others emphasized the bilateral auricular concha (Yu et al., 2017, 2021; Yifei et al., 2022). A different research chose a bilateral approach, specifically on the cymba conchae (Vitello et al., 2023). Another study selected the left tragus (Noé et al., 2020), and yet another preferred the left cymba conchae (Hakon et al., 2020). These variations in stimulation sites highlight the diverse approaches researchers are employing, potentially aiming to maximize the therapeutic efficacy of taVNS.

Among taVNS stimulation in DOC, several studies stimulate the concha bilaterally, whereas quite a few studies usually stimulate the left concha to avoid causing cardiac discomfort. The predominant concern with right-sided or bilateral cervical VNS is its potential to influence cardiac function since the right vagus nerve provides more substantial innervation to the heart (Capilupi et al., 2020). Thus, the traditional approach, especially in implantable VNS devices for epilepsy, is to stimulate the left cervical vagus nerve to mitigate this risk. However, when it comes to the auricular branches, the anatomical and physiological connections might be different (Elamin et al., 2023), which could account for differing side-effect profiles. While some studies have advocated for unilateral left-sided taVNS due to the perceived safety profile, others suggest that bilateral concha stimulation might not present the same cardiac risks as cervical VNS (Ardell et al., 2017; Elamin et al., 2023). However, it’s crucial to approach this notion cautiously, as the evidence is still emerging. Clinical assessments and cardiac monitoring during taVNS can provide essential data regarding this.

### 6.3. Individual variability

#### 6.3.1. Etiology of injury

The etiology of the brain injury may influence the response to taVNS treatment. For example, patients with traumatic brain injuries may have more focal lesions, which could disrupt the structural integrity of the thalamus and brainstem nuclei (Osińska et al., 2022; Yifei et al., 2022). In these cases, taVNS may be more effective in reinstating thalamo-cortical connectivity and promoting consciousness recovery (Yu et al., 2021). On the other hand, anoxic brain injuries often result in diffuse damage to subcortical regions and white matter, which might reduce the effectiveness of taVNS in some patients (Yifei et al., 2022).

#### 6.3.2. Baseline patient characteristics

Baseline patient characteristics, such as the severity of the disorder of consciousness (e.g., UWS vs. MCS), age, and duration since injury, can also influence treatment response (Noé et al., 2020; Zhou et al., 2023). Patients in a MCS may display higher levels of neuroplasticity and reorganization within awareness networks, making them potentially more responsive to brain stimulation interventions like taVNS (Hakon et al., 2020). Additionally, younger patients and those in the early stages of injury may show better treatment responses due to higher neuroplasticity and recovery potential (Yu et al., 2017).

### 6.4. Safety and tolerability

#### 6.4.1. Safety and tolerability of taVNS

TaVNS has generally been reported as a safe and well-tolerated treatment option for various conditions, including DOC, depression, epilepsy, and migraine. The low risk of severe complications associated with taVNS makes it an attractive treatment option for patients with DOC, who may have limited therapeutic options and be more susceptible to complications from invasive treatments (Dong and Feng, 2018). The non-invasive of the intervention, which involves delivering electrical stimulation through the skin overlying the VN, minimizes the risks associated with more invasive procedures such as implanted VNS devices (Yu et al., 2017, 2021; Hakon et al., 2020; Noé et al., 2020; Osińska et al., 2022; Yifei et al., 2022; Zhou et al., 2023). In clinical trials, taVNS has demonstrated good tolerability and compliance, with most patients with DOC able to complete the treatment without significant adverse events (Yu et al., 2017, 2021; Zhou et al., 2023). The side effects of taVNS are generally mild and transient, including skin irritation, itching, or redness at the site of electrode placement, as well as transient dizziness, headache, or nausea (Zhou et al., 2023).

To ensure safety and tolerability, researchers have carefully chosen stimulation parameters for taVNS (Yu et al., 2017, 2021; Hakon et al., 2020; Noé et al., 2020; Osińska et al., 2022; Yifei et al., 2022; Zhou et al., 2023). Current research suggests that typical parameters involve a frequency of 20–25 Hz and a pulse width range of 0.25–1 ms. Studies have shown that the probability of adverse events is extremely low when the stimulation frequency is within the range of 0.5–120 Hz and the pulse width is within 0.02–1 ms. The stimulation intensity generally ranges from 0.2 to 6 mA, but it is usually adjusted according to individual participants. In taVNS studies involving conscious patients, the stimulation current intensity is often set based on the patient’s pain threshold. However, for DOC patients who cannot provide timely feedback, researchers have developed alternative methods, such as setting the stimulation current at 200% of the patient’s perception threshold and calibrating the current to each participant’s minimum perceptual level. Most studies have set the treatment duration to 30 min per session, twice a day.

#### 6.4.2. Individualized treatment considerations

When DOC patients turn into chronic disorders of consciousness, patients may either be in a post-traumatic state or possess severe chronic underlying ailments. However, The relationship between taVNS and varying states of DOC patients remains an emerging area of research. It’s important to note that the modulation of taVNS on Heart Rate Variability (HRV) metrics can differ based on the employed stimulation parameters (De Couck et al., 2017; Geng et al., 2022a). Although some research offers divergent perspectives on taVNS’s influence on HRV metrics, a consensus is emerging that taVNS exhibits considerable potential in ameliorating HRV and augmenting cardiac autonomic regulation in healthy subjects (Geng et al., 2022b). Furthermore, there’s emerging evidence that taVNS could serve as a prophylactic intervention for conditions like pre-diabetes or impaired glucose tolerance (Huang et al., 2014; Li et al., 2018). However, it’s pertinent to mention a significant contraindication: VNS is not recommended for individuals post-splenectomy. Several studies indicate that the spleen plays a critical role in the anti-inflammatory action of the VN (Huston et al., 2006, 2009; Gigliotti et al., 2013; Ji et al., 2014). Once the spleen is removed through splenectomy, the efficacy of VNS in curbing inflammation is notably compromised.

Despite the generally favorable safety profile of taVNS, some prevention strategies should be taken when administering this treatment. Patients with certain medical conditions, such as cardiac arrhythmias or uncontrolled epilepsy, may be at increased risk for complications and should be carefully evaluated before initiating taVNS treatment (Vaseghi et al., 2017; Yuan et al., 2017). Furthermore, individualized treatment protocols should be developed, taking into consideration the patient’s clinical history, the severity of the DOC or other conditions being treated, and any potential contraindications.

## 7. Potential challenges and future research directions

### 7.1. Small sample sizes and lack of control groups

Many existing studies on taVNS in DOC patients have small sample sizes, which may limit the generalizability of the results (Osińska et al., 2022; Yifei et al., 2022). Additionally, some studies lack appropriate control groups, making it difficult to conclusively attribute observed improvements to taVNS treatment (Yu et al., 2017; Noé et al., 2020). Future research should aim to conduct large-scale, randomized controlled trials to provide more robust evidence on the efficacy of taVNS in DOC patients.

### 7.2. Diversity in treatment parameters

There is considerable variability in the treatment parameters used in taVNS research, such as stimulation frequency, intensity, duration, and electrode placement. This variability can make it challenging to compare and synthesize results across studies. Future research should focus on identifying optimal treatment parameters for specific patient populations, as well as investigating potential dose–response relationships and individual factors that may influence treatment outcomes.

### 7.3. Evaluating long-term treatment effects

Many studies have focused on the short-term effects of taVNS treatment, with limited data available on long-term outcomes. It is crucial to assess whether the observed improvements in consciousness and cognitive function persist over time, and whether repeated or prolonged taVNS treatment may yield cumulative benefits. Future studies should include long-term follow-up assessments to better understand the durability and potential lasting effects of taVNS therapy in DOC patients.

### 7.4. Investigating mechanisms of action

While several hypotheses have been proposed to explain the therapeutic effects of taVNS, the precise mechanisms of action remain incompletely understood. Some studies have suggested the involvement of specific brain regions or networks, such as the DMN (Yu et al., 2017), and others have reported changes in CBF (Yu et al., 2021). Future research should aim to elucidate the neural pathways and molecular mechanisms underlying the effects of taVNS, which may ultimately inform the development of more targeted and effective treatment strategies.

In conclusion, within the realm of brain regulation technology for DOC, taVNS technology emerges as a promising avenue for future exploration. Presently, research regarding the clinical outcomes of taVNS and its role in mediating brain–body interplay is nascent. The utilization of taVNS in addressing consciousness impairments is yet to be substantiated by comprehensive evidence-based medical studies, necessitating further scrutiny into its therapeutic modalities and stimulation parameters. As neuroscience research technologies evolve, there will undoubtedly be an enhanced and refined understanding of cerebral structure and functionality.

## Author contributions

LikW: Writing – original draft, Writing – review & editing. FG: Writing – original draft, Writing – review & editing. ZW: Writing – original draft, Writing – review & editing. FL: Conceptualization, Methodology, Writing – original draft. YD: Investigation, Writing – review & editing. MW: Investigation, Writing – review & editing. JW: Conceptualization, Writing – review & editing. YC: Investigation, Writing – review & editing. QY: Investigation, Writing – original draft. LitW: Conceptualization, Investigation, Writing – original draft, Writing – review & editing.
